# Predictors of underage pregnancy among women aged 15–19 in highly prevalent regions of Ethiopia: a multilevel analysis based on EDHS, 2016

**DOI:** 10.1038/s41598-023-27805-y

**Published:** 2023-01-16

**Authors:** Desalegn Anmut Bitew, Yonas Akalu, Yitayeh Belsti, Mengistie Diress, Yibeltal Yismaw Gela, Daniel Gashaneh Belay, Amare Belete Getahun, Bewuketu Terefe, Mihret Getnet

**Affiliations:** 1 Department of Reproductive Health, Institute of Public Health, College of Medicine and Health Sciences, University of Gondar, Gondar, Ethiopia; 2 Department of Human Physiology, School of Medicine, College of Medicine and Health Sciences, University of Gondar, Gondar, Ethiopia; 3 Department of Human Anatomy, School of Medicine, College of Medicine and Health Sciences, University of Gondar, Gondar, Ethiopia; 4 Department of Anesthesia, School of Medicine, College of Medicine and Health Sciences, University of Gondar, Gondar, Ethiopia; 5 Department of Community Health Nursing, School of Nursing, College of Medicine and Health Sciences, University of Gondar, Gondar, Ethiopia; 6 Department of Epidemiology and Biostatics, Institute of Public Health, College of Medicine and Health Sciences, University of Gondar, Gondar, Ethiopia

**Keywords:** Health care, Public health, Epidemiology

## Abstract

Under age (teenage) pregnancy is a pregnancy that occurs under the age of 20 years old. Its magnitude is increasing globally. It is much higher in low-income countries compared to high-income countries. Teenage pregnancy exposed teenagers to various obstetric and perinatal complications. However, its predictors are not well investigated in highly prevalent regions of Ethiopia. Therefore, this study assessed individual and community-level predictors of teenage pregnancy using a multi-level logistic regression model. An in-depth secondary data analysis was performed using the fourth Ethiopian Demographic and Health Survey (EDHS) 2016 data set. A weighted sample of 2397 teenagers was included in the final analysis. Multi co linearity and chi-square tests were checked and variables which did not fulfill the assumptions were excluded from the analysis. Four models were fitted. Variables with p value ≤ 0.2 in the bi-variable multilevel logistic regression were included in the multivariable multilevel logistic regression. The adjusted odds ratio (AOR) with a 95% confidence interval (95% CI) was computed. Variables with a p value of less than 0.05 in the multi-variable multilevel logistic regression were declared as statistically significant predictors. A total of 2397 weighted participants aged from 15 to 19 were involved. About 15% of teenagers were pregnant. Age [17 (AOR = 9.41: 95% CI 4.62, 19.13), 18 (AOR = 11.7: 95% CI 5.96, 23.16), 19 (AOR = 24.75: 95% CI 11.82, 51.82)], primary education (AOR = 2.09: 95% CI 1.16, 3.76), being illiterate (AOR = 1.80: 95% CI 1.19, 2.73), religion [being Muslims (AOR: 2.98:95% CI 1.80, 4.94), being Protestants (AOR = 2.02: 95% CI 1.20, 3.41)], contraceptive non use (AOR = 0.18: 95% CI 0.11, 0.31), a high proportion of family planning demand (AOR = 3.52: 95% CI 1.91, 6.49), and a high proportion of marriage (AOR = 4.30: 95% CI 2.25, 8.21) were predictors of teenage pregnancy. Age, educational status, religion, contraceptive non-use, literacy proportion of marriage and proportion of demand for family planning were the most significant predictors of teenage pregnancy. The ministry of education shall focus on universal access to education to improve female education. The government should work in collaboration with religious fathers to address reproductive and sexual issues to decrease early marriage and sexual initiation. Especial attention should be given to teenagers living in a community with a high proportion of marriage.

## Introduction

Adolescence is a developmental stage during which physical, emotional, mental, and societal changes occur^1^. Since adolescents are unaware of sexuality as well as sexual and reproductive health services, they are victims of teenage pregnancy and associated complications^2–4^. According to the World Health Organization (WHO), teenage pregnancy is a pregnancy that occurs under the age of 20 years old. It usually refers to pregnancy among teens between the ages of 15–19^5^.

Although adolescent motherhood is becoming more common in both developed and underdeveloped countries, it is substantially higher in low-income countries^4,6,7^. Only seven nations i.e. Bangladesh, Brazil, the Democratic Republic of Congo, Ethiopia, India, Nigeria, and the United States account for about half of all teenage births^8–10^. In underdeveloped countries, an estimated 21 million adolescent girls become pregnant, every year^11^.

The overall pooled prevalence of teenage pregnancy in Africa and sub-Saharan Africa was 18.8% and 19.3% respectively^12^. According to EDHS 2016, 13% of adolescent girls have begun childbearing. However, it varies greatly across regions. it was 3% in Addis Ababa, 8% in Amhara, 11% in SNNPR, 12% in Tigray, 13% in Dire Dawa, 14% in Benishangul Gumuz, 16% in Gambela, 17% in Harari and Oromiya, 19% in Somali and 23% in Afar^13^.different local studies in Ethiopia shows that, the magnitude of teenage pregnancy was 28.6% in Northeast Ethiopia^14^, 30.2% in eastern Ethiopia^15^, 25.4% in Farta Woreda^16^ and 7.7% in Arba Minch town^17^.

Annually, about 69.64% of unsafe abortions occur among adolescent girls. This contributes to maternal death, maternal morbidity, and long-term health complications^18^. Teenage pregnancy is associated with several complications such as preterm labor, intrauterine growth retardation, low birth weight^19–21^, neonatal death, obstructed labor, fistula, and eclampsia^7,20,22^, increased maternal mortality and morbidity^9,21^, preterm premature rupture of membranes, gestational hypertension, and preeclampsia, poor intrauterine growth, and stillbirths^2,21,23^, unsafe abortion, and sexually transmitted infections. Teen girls are twice more likely to die during pregnancy and childbirth compared to women in twenties^24^.

The most commonly reported predictors of teenage pregnancy are low socioeconomic status and educational level^7,25–27^, lack of knowledge of sexuality, ineffective use of modern contraceptives, cultural obedience, and peer influence^26–29^, not communicating with parents on reproductive health issues^8^, early sexual initiation^27,30^, early marriage^27,30^, residence, living in a community with a lower proportion of contraceptive users^27,30^, gender inequity, and physical/sexual violence^27^.

As reducing teenage pregnancy and maternal mortality are among sustainable development goals (SDGs), it is being implemented by the Ethiopian government and other development partners^31^. Though, Ethiopian federal ministry of health (FMOH) devised a variety of initiatives and set goals to lower the rate of teenage pregnancy from 13 to 3%^32^, the rate in Ethiopia remains high^33^.

Although various studies have been conducted in Ethiopia on this topic, the majorities of them were local, had a limited sample size, and employed simple analytical models that ignored community-level predictors of teenage pregnancy. Our study differs from the two national multilevel investigations^30,34^, by first, our research was conducted in Ethiopia's high-prevalent regions, using individual record (IR) data; second, hearing family planning messages in the media, family planning demand at the individual and community level, the proportion of marriage in the community, and literacy are included in the analysis. All of which had not been addressed in previous studies. The existing studies did not answer what are the determinants of teenage pregnancy in the highly prevalent regions. High prevalent regions may have different factors than the general population of Ethiopia.

It is critical to understand the predictors of teenage pregnancy to prevent the medical, social, and economic consequences. Therefore, this study aimed to assess individual and community-level predictors of teenage pregnancy using a multi-level logistic regression model.

### Methods and materials

#### Study area, setting

This study was conducted in high prevalent regions (high rates of teen pregnancy) of Ethiopia [Tigray, Afar, Oromiya, Somalia, Benishangul-Gumuz, Southern Nations Nationalities and Peoples' Region (SNNPR), Gambela, and Harari)]. Ethiopia. It has a total estimated 118,977,453 population^35^. Though Ethiopia is making the most progress in Sub Saharan Africa (SSA) in terms of assuring access to education, it still has obstacles, including low primary completion rates, a drop in secondary enrolment rates (30.7%), and poor educational quality at all levels^36^.

### Study design, period and sampling

An in-depth secondary data analysis was performed using the fourth Ethiopian Demographic and Health survey (EDHS) 2016 data set. The 2016 EDHS was done in nine regional states and two administrative cities using cross-sectional study design. The EDHS was based on 645 enumeration areas. Details of the EDHS methodology are found on the EDHS reports^13^. The EDHS has been conducted every 5 years to provide health and health-related indicators in Ethiopia.

### Data source and study population

We have used the individual record (IR) data set of EDHS 2016 for this study. The data was accessed from the measure DHS website (http://www.measuredhs.com). Totally, 509 enumeration areas (EAs) were included in this study. A total of 2711 younger women aged 15–19 years were interviewed about teenage pregnancy at the time of the survey after weighting, a total of 2397 teenagers were included in the final analysis. All the frequencies and percentages in the result section were weighted.

### Variables and measurement

The outcome variable was teenage pregnancy. It was dichotomized as (yes/no). A woman was considered as experiencing teenage pregnancy if her age was from 15 to 19 and had a birth or was pregnant at the time of the interview. The independent variables were grouped under individual-level variables and community level variables. Individual level variables include age, marital status, educational status, literacy, religion, working status, wealth index, age at marriage, age at first sex, media exposure, contraceptive use, demand to family planning, hearing family planning, messages on mass media, sex of household head and age of household head. Whereas community level variables include residence, community wealth, community literacy, community education, community media exposure, community family planning demand, community percentage of marriage, community family planning message transfer and community working status.

#### Individual level variables

##### Age at first marriage

The respondent's age at first marriage is the age at which she began living with her first spouse/partner. It was divided into three groups. "Married before the age of 15", "married between the ages of 15 and 17", and "not married before the age of 18". Those who were not married before the age of 18 include those who were married after the age of 18 and those who were not married during their lifetime.

##### Sexual experience

Was categorized into four as “never had sex, "active before age 15", "active between ages 15 and 17", and "active at age 18 and above".

##### Educational status of women

This variable is divided into three categories: "no education", "primary", and "secondary and higher education".

##### Working status

This has been categorized as "Yes" and "No" in the 2016 EDHS.

##### Media exposure

Watching television (TV), listening to the radio and reading newspapers both less than once a week and at least once a week were considered to measure exposure to media.

##### Wealth index

Within the dataset, the wealth index was presented as Poorest, Poorer, Middle, Richer, and Richest. In this study, a new variable was generated with three categories as "Poor", "Middle" and "Rich" by merging poorest with poorer and richest with richer.

##### Religion

In the 2016 EDHS, religion was categorized as Orthodox, Muslim, Protestant, Catholic, traditional followers and others. In this study, the former three were encoded independently and Catholic and traditional religion followers were merged into the "others" category.

##### Hearing family planning messages

This variable was generated from the four sources of messages related to family planning "Heard family planning on radio last few months", "Heard family planning on radio last few months", and "Heard family planning on radio last few months". These were measured as "yes or no" in the 2016 EDHS. In this study, participants were considered to hear family planning messages if they said "yes" at least for one of the sources.

##### Literacy

In the EDHS 2016 this variable was recorded as cannot read at all, Able to read only parts of a sentence, Able to read the whole sentence, having No card with required language and being Blind/visually impaired. In this study, it was coded as literate (those able to read) and illiterate (the rest categories).

#### Community level variables

Community-level variables were computed by aggregating the individual level women's characteristics into clusters. Then the proportion was calculated by dividing subcategories by the total. Distributions of the proportion of aggregate variables were checked using the Shapiro–Wilk normality test and were not normally distributed. Therefore, these aggregate variables were categorized using the median value. A total of eight community variables were generated. A residence was taken as a community-level variable. Therefore a total of nine community variables were tested (residence, community wealth, community literacy, community education, community family planning demand, community media exposure, community level marriage, community family planning message transfer, and community working status).

### Data processing and analysis

Descriptive statistics including frequencies, medians, and percentages were produced once the data had been cleaned. Stata version 14.0 was used to analyze the data. Sampling weights were used to account for the sample's non-proportional strata allocation and non-responses. Individuals were nested inside communities in the EDHS data, and the intra-class correlation coefficient (ICC) was 29.33%. To evaluate the independent (fixed) effects of the explanatory variables as well as the community-level random effects on teenage pregnancy, a two-level mixed-effects logistic regression model was used. Multi-co linearity was checked and variables with a variance inflation factor greater than 10 were excluded. Some variables which did not fulfill the chi-square test were also excluded from the analysis.

This study used four models [Model 0 (no factors), Model 1 (individual level factors), Model 2 (only community-level factors), and Model 3 (both individual and community-level factors)]. The multivariable multilevel logistic regression analysis includes variables with a p value of 0.2 from the bi-variable multilevel logistic regression analysis. The Adjusted Odds Ratio (AOR) with a 95% confidence interval (95% CI) was computed. Variables with a p value of less than 0.05 in the multi-variable multilevel logistic regression analysis in the final model were declared as statistically significant predictors of the outcome variable.

## Results

In this study, a total of 2397 weighted adolescent girls were participated. The mean (± SD) age of study participants was 16.8 (± 1.36). One thousand eight hundred seven (78.6%) were ever married. The majority of study participants, 1577 (65.8%) had primary education. Only 4.88% (117) of participants were contraceptives users (Table 1).

**Table 1:** Individual level characteristics and teenage pregnancy distribution (n = 2397), Ethiopia 2016.

Variables	Categories	Percentage (%)	Teenage px (%)	
			No (%)	Yes (%)
Age	15	513 (21.4)	502 (20.9)	11 (0.5)
	16	504 (21)	481 (20)	23 (1)
	17	460 (19.2)	381 (15.9)	79 (3.3)
	18	646 (26.9)	505 (21)	141 (5.9)
	19	274 (11.5)	177 (7.4)	97 (4.1)
Marital status	Ever married	1887 (78.6)	1872 (78)	15 (.6)
	Never married	510 (21.4)	173 (7.3)	337 (14.1)
Educational status	No education	363 (15.1)	255 (10.6)	108 (4.5)
	Primary	1577 (65.8)	1359 (56.7)	218 (9.1)
	Secondary and above	457 (19.1)	431 (18)	26 (1.1)
Literacy	Illiterate	843 (35.2)	636 (26.6)	207 (8.6)
	Literate	1554 (64.8)	1409 (58.8)	145 (6.0)
Religion	Orthodox	606 (25.3)	557 (23.2)	49 (2.1)
	Muslim	920 (38.4)	711 (29.7)	208 (8.7)
	Protestant	828 (34.5)	741 (30.9)	87 (3.6)
	Others	43 (1.8)	36 (1.5)	7 (0.3)
Working status	Yes	573 (23.9)	499 (20.8)	74 (3.1)
	No	1824 (76.1)	1547 (64.4)	277 (11.7)
Wealth index	Poor	818 (34.1)	638 (26.6)	180 (7.5)
	Middle	488 (19.1)	382 (15.9)	76 (3.2)
	Rich	1121 (46.8)	1026 (42.8)	95 (4.0)
Age at marriage	Never married	1887 (78.7)	1872 (78.1)	15 (0.6)
	< 15	121 (5.2)	21 (0.9)	100 (4.3)
	15–17	330 (13.8)	106 (4.5)	224 (9.3)
	18 and above	59 (2.4)	46 (1.9)	13 (0.5)
Age at first sex	Never had sex	1835 (76.6)	1835 (76.6)	0 (0)
	< 15	120 (5.0)	35 (1.5)	85 (3.5)
	15–17	382 (16)	128 (5.4)	254 (10.6)
	18 and above	60 (2.4)	47 (1.9)	13 (0.5)
Media exposure	No	1223 (50.6)	990 (41.3)	223 (9.3)
	Yes	1184 (49.4)	1056 (44)	128.1 (5.4)
Contraceptive use	No	2280 (95.12)	1996 (83.26)	284 (11.86)
	Yes	117 (4.88)	50 (2.07)	67 (2.81)
Demand to FP	No demand	1837 (76.65)	1836 (76.62)	1 (0.03)
	demand	560 (23.35)	209 (8.72)	351 (14.63)
Hearing FP messages on mass medias	No	1331 (55.5)	1118 (46.7)	213 (8.8)
	Yes	1066 (44.5)	927 (38.7)	139 (5.8)
Sex of HHH	Male	1786 (74.5)	1503 (62.7)	283 (11.8)
	Female	611 (25.5)	543 (22.6)	68 (2.9)
Age of HHH	15–24	205 (8.5)	95 (3.96)	110 (4.6)
	25–34	258 (10.8)	157 (6.6)	101 (4.2)
	35–44	472 (19.7)	448 (18.7)	24 (1.0)
	45–54	602 (25.1)	556 (23.2)	46 (1.9)
	55–64	497 (20.7)	465 (19.4)	32 (1.3)
	65–74	260 (10.86)	232 (9.68)	28 (1.18)
	75–84	71 (2.96)	63 (2.65)	8 (0.31)
	85–95	32 (1.3)	29 (1.2)	3 (0.1)

*FP*: family planning, *HHH*: household head*.*

One thousand nine hundred fifty-five (81.5) of the study participants were rural dwellers. Nearly half (47.4%) of the participants were from communities with a high proportion of poorness. One thousand three hundred (55.59%) participants were from communities with a low proportion of above secondary education. About 47% (1132) of teenagers were from communities with a high proportion of early marriage (Table 2).

**Table 2:** Community level characteristics and teenage pregnancy distribution (n = 2397), Ethiopia 2016.

Variables	Categories	Percentage (%)	Teenage px (%)	
			No (%)	Yes (%)
Residence	Urban	442 (18.5)	413.5 (17.3)	28.79 (1.2)
	Rural	1955 (81.5)	1632 (68.1)	323 (13.4)
Community wealth	Low proportion of poor	1260 (52.6)	1141 (47.59)	119 (4.98)
	High proportion of poor	1137 (47.4)	905 (37.7)	232 (9.7)
Community literacy	Low proportion of literacy	1152 (48.04)	909 (37.92)	242.5 (10.12)
	High proportion of literacy	1245 (51.96)	1136 (47.41)	109 (4.55)
Community education	Low proportion of above secondary education	1332 (55.59)	1074 (44.81)	258 (10.78)
	High proportion of above secondary education	1064 (44.41)	971 (40.52)	93 ( 3.89)
Community media exposure	Low proportion of media exposure	1275 (53.18)	1141 (47.62)	134 (5.56)
	High proportion of media exposure	1122 (46.82)	904 (37.71)	218 (9.11)
Community FP demand	Low proportion of FP demand	1272 (53.06)	1232 (51.4)	40 (1.66)
	High proportion of FP demand	1125 (46.94)	813 (33.93)	312 (13.01)
Community level marriage	Low proportion of marriage	1265 (52.76)	1232 (51.41)	32.49 (1.36)
	High proportion of marriage	1132 (47.24)	813 (33.92)	319 (13.31)
Community FP message transfer	Low proportion of FP message transfer	1248 (52.05)	1022 (42.62)	226 (9.43)
	High proportion of FP message transfer	1149 (47.95)	1024 (42.71)	125 (5.24)
Community working status	Low proportion of working status	1119 (46.69)	921 (38.42)	198 (8.27)
	High proportion of working status	1278 (53.31)	1124 (46.91)	154 (6.40)

*Px*: pregnancy, *FP*: family planning.

### Predictors of teenage pregnancy

Multilevel mixed effect logistic regression model was employed. The measures of variations or random effects were reported using intra-class correlation (ICC), a proportional change in variance (PCV), and Median Odds Ratio (MOR). PCV was computed as: $PCV=\frac{Vnull -VA}{V null}$^37^ and MOR is a measure of unexplained cluster heterogeneity and it was computed as: $MOR={e}^{0.95\sqrt{VA}}$^37^ where “VA” represents the area or cluster level variance. The ICC was used to show how much the observation within one cluster resembled each other and it was generated directly from each model using “estat ICC” command following regression. The model comparison was done using the likelihood ratio. The model with highest likely hood ratio was selected, and (Table 3).

**Table 3:** Random effect and model of two-level mixed effect logistic regression models predicting Teenage pregnancy, Ethiopia 2016.

Parameters	Null model	Model III (final model)
Community variance (SE)	1.37 (.27)	0.099 (.07)
ICC	29.33%	2.95%
PCV	Reference	92.7%
MOR	3.02	0.81
Model fitness statistics (Log likelihood)	− 920.02	− 800.02

*ICC*: intra class correlation, *PCV*: proportional change in variance, *MOR*: median odds ratio

In the multilevel mixed effect multivariable logistic regression model, the age of respondents, education, literacy, religion, contraceptive use, community level demand for family planning, and proportion of marriage in the community were statistically significant predictors of teenage pregnancy.

The odds of experiencing teenage pregnancy at 17 years was 9.41 (AOR = 9.41: 95% CI 4.62, 19.13) times higher than teenagers at 15 years. The odds of experiencing teenage pregnancy at 18 years was 11.7 (AOR = 11.7: 95% CI 5.96, 23.16) times higher than teenagers at 15 years. The odds of experiencing teenage pregnancy at 19 years was 24.75 (AOR = 24.75: 95% CI 11.82, 51.82) times higher compared to teenagers at 15 years. The odds of experiencing teenage pregnancy among teenagers who have primary education was 2.09 (AOR = 2.09: 95% CI 1.16, 3.76) times higher compared to teenagers with secondary and above education. Illiterate teenagers have 1.8 (AOR = 1.80: 95% CI 1.19, 2.73) times higher odds of experiencing teenage pregnancy compared to literate teenagers. The odds of experiencing teenage pregnancy among Muslims and Protestants were 2.98 (AOR: 2.98:95% CI 1.80, 4.94) and 2.02 (AOR = 2.02: 95% CI 1.20, 3.41) times higher respectively compared to orthodox. The odds of experiencing teenage pregnancy among contraceptive non-users were reduced by 82% (AOR = 0.18: 95% CI 0.11, 0.31) compared to contraceptive users. Teenagers living in a community with a high proportion of family planning demand have 3.52 (AOR = 3.52: 95% CI 1.91, 6.49) times increased odds of teenage pregnancy compared to their counterparts. Teenagers living in a community with a high proportion of marriage have 4 0.30 (AOR = 4.30: 95% CI 2.25, 8.21) times increased odds of teenage pregnancy compared to their counterparts (Table 4).

**Table 4:** Individual and community-level factors associated with teenage pregnancy, EDHS 2016 (n = 2397).

Individual and community Level characteristics		COR (95% CI)	Final model AOR (95% CI)
Age	15	1	1
	16	2.07 (0.92, 4.64)	1.75 (0.81, 3.80)
	17	12.43 (5.92, 26.10)	9.41 (4.62,19.13)**
	18	17.71 (8.65, 36.2)	11.7 (5.96, 23.16)**
	19	37.15 (17.53, 78.74)	24.75 (11.82, 51.82)**
Educational status	No education	5.50 (3.18, 9.51)	1.96 (0.92, 4.17)
	Primary	2.23 (1.38, 3.59)	2.09 (1.16, 3.76)*
	Secondary and above	1	1
Literacy	Illiterate	2.94 (2.21, 3.91)	1.80 (1.19, 2.73)*
	Literate	1	**1**
Religion	Orthodox	1	1
	Muslim	3.35 (2.12, 5.30)	2.98 (1.80, 4.94)**
	Protestant	1.53 (0.94, 2.49)	2.02 (1.20, 3.41)*
	Others*	2.24 (0.61, 8.18)	1.52 (0.40, 5.71)
Wealth	Poor	2.25 (1.60, 3.18)	1.31 (0.83, 2.08)
	Middle	1.86 (1.26, 2.74)	1.48 (0.92, 2.38)
	Rich	1	1
Media exposure	No	1.55 (1.16, 2.07)	1.28 (0.88, 1.87)
	Yes	1	1
Hearing FP messages	No	1	1.03 (0.70, 1.50)
	Yes	1.14 (0.85, 1.54)	1
Contraceptive use	No	0.06 (0.04, 0.10)	0.18 (0.11, 0.31)**
	Yes	1	1
Sex of HHH	Male	1.28 (0.92, 1.78)	0.98 (0.68, 1.43)
	Female	1	1
Residence	Urban	1	1
	Rural	2.9 (1.56, 5.30)	1.33 (0.67, 2.61)
Community wealth	Low proportion of poor	1	1
	High proportion of poor	2.83 (1.87, 4.30)	1.06 (0.66, 1.69)
Community literacy	Low proportion of literacy	3.26 (2.17, 4.90)	1.02 (0.63, 1.66)
	High proportion of literacy	1	1
Community education	Low proportion of above secondary education	2.59 (1.72, 3.92)	0.73 (0.46, 1.17)
	High proportion of above secondary education	1	1
Community media exposure	Low proportion of media exposure	2.17 (1.44, 3.26)	0.78 (0.48, 1.25)
	High proportion of media exposure	1	1
Community FP demand	Low proportion of FP demand	1	1
	High proportion of FP demand	13.06 (8.80, 19.37)	3.52 (1.91, 6.49)**
Community marriage	Low proportion of marriage	1	1
	High proportion of marriage	15.47 (10.36,23.12)	4.30 (2.25, 8.21)**
Community FP message	Low proportion of FP message transfer	1.71 (1.14, 2.59)	1.20 (0.77, 1.89)
	High proportion of FP message transfer	1	1
Community working status	Low proportion of working status	1.35 (0.89, 2.03)	1.00 (0.69, 1.47)
	High proportion of working status	1	1

Significant values are in [bold].

*FP*: family planning,* HHH*: household head, *px*: pregnancy.

## Discussion

The age of respondents, educational status, literacy, contraceptive use, religion, family planning demand at the community level and proportion of marriage in the community were found to be significant predictors of teenage pregnancy. Regarding age, teenagers aged 17, 18, and 19 had higher odds of experiencing teenage pregnancy than 15 years olds teenagers. This is similar to other studies done in Ethiopia^8,38,39^ and Kenya^26^. The possible explanation for this finding could be, as teenagers get older, the probability of being sexually active and getting married will be increased. Consequently, the chance of getting pregnant and childbirth will also increase. This implies that sexual and reproductive health programs (SRH) should be designed to focus on late teenagers.

Level of education was also found to be a predictor of teenage pregnancy. In this study, teenagers with primary education have higher odds of teenage pregnancy compared to teenagers with secondary education and above. This finding is in agreement with the findings of studies from Ethiopia^38,40^, Malawi^27^, Australia^41^, East Africa countries^42^, Kenya^26,43^, and European Union countries^25^ which states better education results a fall in teenage pregnancy^44^. The possible explanation could be, teenagers with higher levels of education are accessible to relevant information and would have better knowledge of reproductive and sexual health such as the risk of unprotected sex, consequences of early pregnancy and preventive measures^27^. This implies that teenagers should be educated to at least secondary education.

Surprisingly, contraceptive use increases the odds of teenage pregnancy in our study. Though our study finding is contradicted with many scientific pieces of evidence from Ethiopia^30,38–40^, Malawi^27^, Uganda^45^, it is supported by study finding from Kenya^26^. One study concludes that family planning has an ambiguous impact on teen pregnancy and no evidence that the provision of family planning reduces teenage pregnancy^46^. The possible explanation for this finding could be poor quality and efficacy of birth control methods and services, and improper use of contraceptives can result in unwanted pregnancies. In our study, most of the contraceptive non-users are not in a marital union or are sexually active and have no risk of pregnancy.

This study also shows that there is a difference in the rate of teenage pregnancy among different religions. Being Muslim and protestant increases the odds of teenage pregnancy compared to being orthodox. Regarding Muslims, similar findings were documented in Bangladesh^47^, Sub Saharan Africa^48^ and Nigeria^49^ which shows that women in the Islamic religion tended early pregnancy and childbirth than women in other religions. The possible explanation can be due to the likely hood of Muslims marrying at an age less than 15 years^47,50^. Concerning being protestant, there was supportive evidence in Ghana which shows the liberal attitude of women towards sexual activity increases the likely hood of women's premarital sexual intercourse and underage pregnancy^51^. The difference in teenage pregnancy among different religions may also be explained by the difference in attitudes, norms and beliefs about birth control and the value of children among different religions^52,53^. This implies that there should be collaboration with religious fathers to prevent teenage pregnancy and its complications.

Another predictor of teenage pregnancy was the proportion of marriage in the community. This study revealed that teenager from a community with a high proportion of marriage has higher odds of experiencing teenage pregnancy. This finding was supported by findings from Uganda^45,54^ and Nigeria^55,56^. The percentage of women in sexual union and frequency of sexual intercourse is the most important proximal determinants of fertility^57^. For pregnancy to occur sexual intercourse is a must and marriage increases the frequency of sexual contact. This implies that the legal age of marriage should be strictly followed to prevent early marriage and to reduce the percentage of marriage in the community.

The proportion of family planning demand in the community was also a predictor of teenage pregnancy. This shows that teenagers in a community with a high proportion of family planning have higher odds of teenage pregnancy. This finding was supported by evidence from Washington^58^. The possible explanation is that, larger proportion of family planning demand in adolescents is unmet need for contraception resulting in unintended pregnancies^58^. This implies that the family planning needs of teenagers should be met through expanded SRH services. Literacy was also found to predict the rate of teenage pregnancy. In this study, illiterate teenagers had higher odds of teenage pregnancy than literate. This finding was supported by evidence from the united state^59^. A possible explanation could be literate teenagers have a better understanding of reproductive and sexual issues through reading printed materials like newspaper magazines and books.

### Strengths and limitations

We believe our study had several strengths such as we used nationwide data with better statistical power and used multilevel approaches. However, using secondary data limit the researcher to measure all possible determinants like culture and tradition-related factors. The accuracy of the data could be affected by recall bias since the source of the data was self-report.

### Ethical approval and consent to participate

Since this study was conducted based on EDHS data which is available by request from the measure DHS website (http://www.measuredhs.com), ethics approval was not required for this study. All methods of this research were done following the declaration of Helsinki. The data was collected anonymously during the survey and used anonymously during the current analysis.

## Conclusion and recommendations

This study identified potential predictors of teenage pregnancy. As a result, the most significant predictors of teenage pregnancy were socio demographic factors such as age, educational status, religion, literacy, proportion of marriage and some family planning related factors such as contraceptive non-use and proportion of demand for family planning factors. Literacy, proportion of marriage and proportion of demand for family planning were new findings from the previous studies. The ministry of education shall focus on universal access to education to improve female education. The government should work in collaboration with religious fathers to address reproductive and sexual issues to decrease early marriage and sexual initiation. Especial attention should be given to teenagers living in a community with a high proportion of marriage. This study strongly recommends to future researchers that, to use primary data and better to include cultural and tradition related factors.

## Data Availability

The dataset supporting the conclusions of this article is available in the measure DHS website (http://www.measuredhs.com) and the extracted data is available with the corresponding author.

## Abbreviations

AOR: Adjusted odds ratio
COR: Crude odds ratio
CI: Confidence interval
EDHS: Ethiopia demographic and health survey
ICC: Intra cluster correlation coefficient
MOR: Median odds ratio
PCV: Proportional change in variance
SRHS: Sexual and reproductive health services

## References

[1] Blakemore S-J (2019). Adolescence and mental health. Lancet.

[2] Pergialiotis V, Vlachos D-E, Gkioka E, Tsotra K, Papantoniou N, Vlachos G (2015). Teenage pregnancy antenatal and perinatal morbidity: Results from a tertiary centre in Greece. J. Obstet. Gynaecol..

[3] Gupta N, Kiran U, Bhal K (2008). Teenage pregnancies: Obstetric characteristics and outcome. Eur. J. Obstetr. Gynecol. Reprod. Biol..

[4] Neal S, Matthews Z, Frost M, Fogstad H, Camacho AV, Laski L (2012). Childbearing in adolescents aged 12–15 years in low resource countries: A neglected issue. New estimates from demographic and household surveys in 42 countries. Acta Obstetr. Gynecol. Scand..

[5] World Health Organization (WHO). Adolescent Pregnancy. 2004.

[6] Whitworth M, Cockerill R, Lamb H (2017). Antenatal management of teenage pregnancy. Obstet. Gynaecol. Reprod. Med..

[7] Ayuba II, Gani O (2012). Outcome of teenage pregnancy in the Niger Delta of Nigeria. Ethiop. J. Health Sci..

[8] Ayele BGK, Gebregzabher TG, Hailu TT, Assefa BA (2018). Determinants of teenage pregnancy in Degua Tembien District, Tigray, Northern Ethiopia: A community-based case-control study. PLoS One.

[9] Caffe S, Plesons M, Camacho AV, Brumana L, Abdool SN, Huaynoca S (2017). Looking back and moving forward: Can we accelerate progress on adolescent pregnancy in the Americas?. Reprod. Health.

[10] Islam MM, Islam MK, Hasan MS, Hossain MB (2017). Adolescent motherhood in Bangladesh: Trends and determinants. PLoS One.

[11] Darroch, J. E., Woog, V., Bankole, A., & Ashford, L. S. Adding it up: Costs and benefits of meeting the contraceptive needs of adolescents. 2016.

[12] Kassa GM, Arowojolu A, Odukogbe A, Yalew AW (2018). Prevalence and determinants of adolescent pregnancy in Africa: A systematic review and meta-analysis. Reprod. Health.

[13] Central Statistical Agency-CSA/Ethiopia, ICF. Ethiopia Demographic and Health Survey 2016. Addis Ababa, Ethiopia: CSA and ICF, 2017.

[14] Ayanaw Habitu Y, Yalew A, Azale BT (2018). Prevalence and factors associated with teenage pregnancy, Northeast Ethiopia, 2017, a cross-sectional study. J. Pregnancy.

[15] Mezmur H, Assefa N, Alemayehu T (2021). Teenage pregnancy and its associated factors in eastern Ethiopia: A community-based study. Int. J. Women's Health.

[16] Kassa BG, Belay HG, Ayele AD (2021). Teenage pregnancy and its associated factors among teenage females in Farta woreda, Northwest, Ethiopia, 2020: A community-based cross-sectional study. Popul. Med..

[17] Mathewos S, Mekuria A (2018). Teenage pregnancy and its associated factors among school adolescents of Arba Minch Town, Southern Ethiopia. Ethiop. J. Health Sci..

[18] https://www.who.int/news-room/fact-sheets/detail/adolescent-pregnancy. 2020.

[19] Rasheed S, Abdelmonem A, Amin M (2011). Adolescent pregnancy in upper Egypt. Int. J. Gynecol. Obstet..

[20] Wu W-Y, Li C-R, Kuo C-P, Chiang Y-C, Lee M-C (2016). The growth and development of children born to adolescent mothers in Taiwan. Ital. J. Pediatr..

[21] Kumar A, Singh T, Basu S, Pandey S, Bhargava V (2007). Outcome of teenage pregnancy. Indian J. Pediatr..

[22] Indarti J, Al Fattah AN, Dewi Z, Hasani RDK, Mahdi FAN, Surya R (2020). Teenage pregnancy: Obstetric and perinatal outcome in a tertiary centre in Indonesia. Obstetr. Gynecol. Int..

[23] Bateman BT, Simpson LL (2006). Higher rate of stillbirth at the extremes of reproductive age: A large nationwide sample of deliveries in the United States. Am. J. Obstet. Gynecol..

[24] Omar K, Hasim S, Muhammad NA, Jaffar A, Hashim SM, Siraj HH (2010). Adolescent pregnancy outcomes and risk factors in Malaysia. Int. J. Gynecol. Obstet..

[25] Imamura M, Tucker J, Hannaford P, Da Silva MO, Astin M, Wyness L (2007). Factors associated with teenage pregnancy in the European Union countries: A systematic review. Eur. J. Pub. Health.

[26] Were M (2007). Determinants of teenage pregnancies: The case of Busia District in Kenya. Econ. Hum. Biol..

[27] Kaphagawani NC, Kalipeni E (2017). Sociocultural factors contributing to teenage pregnancy in Zomba district, Malawi. Glob. Public Health.

[28] Abebe AM, Fitie GW, Jember DA, Reda MM, Wake GE (2020). Teenage Pregnancy and Its Adverse Obstetric and Perinatal Outcomes at Lemlem Karl Hospital, Tigray, Ethiopia, 2018. Biomed. Res. Int..

[29] Fouelifack FY, Tameh TY, Mbong EN, Nana PN, Fouedjio JH, Fouogue JT (2014). Outcome of deliveries among adolescent girls at the Yaoundé central hospital. BMC Pregnancy Childb..

[30] Birhanu BE, Kebede DL, Kahsay AB, Belachew AB (2019). Predictors of teenage pregnancy in Ethiopia: A multilevel analysis. BMC Public Health.

[31] Nations U (2015). Transforming Our World: The 2030 Agenda for Sustainable Development.

[32] Ababa, A. Federal democratic republic of Ethiopia ministry of health. Ethiopia: Postnatal Care. 2003.

[33] Central Statistical Agency CSAE, Icf. Ethiopia Demographic and Health Survey 2016. Addis Ababa, Ethiopia: CSA and ICF, 2017.

[34] Kefale B, Yalew M, Damtie Y, Adane B (2020). A Multilevel analysis of factors associated with teenage pregnancy in Ethiopia. Int. J. Women's Health.

[35] https://www.worldometers.info/world-population/ethiopia-population/. Accessed December 2021.

[36] GEM Report. https://gemreportunesco.wordpress.com/2019/07/12/ethiopia-is-making-the-fastest-progress-in-primarycompletion-in-sub-saharan-africa-how/. Accessed 5 Sept.

[37] Belay DG, Getnet M, Akalu Y, Diress M, Gela YY, Getahun AB (2022). Spatial distribution and determinants of bottle feeding among children 0–23 months in Ethiopia: Spatial and multi-level analysis based on 2016 EDHS. BMC Pediatr..

[38] Alemayehu T, Haider J, Habte D (2010). Determinants of adolescent fertility in Ethiopia. Ethiop. J. Health Dev..

[39] Ayanaw Habitu Y, Yalew A, Azale BT (2018). Prevalence and factors associated with teenage pregnancy, Northeast Ethiopia, 2017: A cross-sectional study. J. Pregnancy.

[40] Geda YF (2019). Determinants of teenage pregnancy in Ethiopia: A case–control study, 2019. Curr. Med. Issues.

[41] Maravilla JC, Betts KS, Cruz CC, Alati R (2017). Factors influencing repeated teenage pregnancy: A review and meta-analysis. Am. J. Obstetr. Gynecol..

[42] Wado YD, Sully EA, Mumah JN (2019). Pregnancy and early motherhood among adolescents in five East African countries: A multi-level analysis of risk and protective factors. BMC Pregnancy Childb..

[43] Omoro T, Gray SC, Otieno G, Mbeda C, Phillips-Howard PA, Hayes T (2018). Teen pregnancy in rural western Kenya: A public health issue. Int. J. Adolesc. Youth.

[44] Girma S, Paton D (2015). Is education the best contraception: The case of teenage pregnancy in England?. Soc. Sci. Med..

[45] Ochen AM, Chi PC, Lawoko S (2019). Predictors of teenage pregnancy among girls aged 13–19 years in Uganda: A community based case-control study. BMC Pregnancy Childb..

[46] Paton D (2002). The economics of family planning and underage conceptions. J. Health Econ..

[47] Sarkar P (2010). Determinants of age at first birth in Bangladesh. J. Mod. Math. Stat..

[48] Yakubu I, Salisu WJ (2018). Determinants of adolescent pregnancy in sub-Saharan Africa: A systematic review. Reprod. Health.

[49] Fagbamigbe AF, Idemudia ES (2016). Survival analysis and prognostic factors of timing of first childbirth among women in Nigeria. BMC Pregnancy Childb..

[50] Ali M, Alauddin S, Maniruzzaman M, Islam SMS (2021). Determinants of early age of mother at first birth in Bangladesh: A statistical analysis using a two-level multiple logistic regression model. J. Public Health.

[51] Addai I (2000). Religious affiliation and sexual initiation among Ghanaian women. Rev. Religious Res..

[52] Gyimah SO (2003). A cohort analysis of the timing of first birth and fertility in Ghana. Popul. Res. Policy Rev..

[53] Taggart T, Gottfredson N, Powell W, Ennett S, Chatters LM, Carter-Edwards L (2018). The role of religious socialization and religiosity in African American and Caribbean black adolescents’ sexual initiation. J. Relig. Health.

[54] Manzi F, Ogwang J, Akankwatsa A, Wokali OC, Obba F, Bumba A (2018). Factors associated with teenage pregnancy and its effects in Kibuku Town Council, Kibuku District, Eastern Uganda: A cross sectional study. Primary Health Care.

[55] Envuladu EA, Agbo HA, Ohize VA, Zoakah AI (2014). Determinants and outcome of teenage pregnancy in a rural community in Jos, Plateau State, Nigeria. Sub-Saharan Afr. J. Med..

[56] Ayoade MA (2021). Spatiotemporal patterns of teenage pregnancy in Nigeria: Evidence from the 2008, 2013 and 2018 NDHS. Pap. Appl. Geogr..

[57] Laelago T, Habtu Y, Yohannes S (2019). Proximate determinants of fertility in Ethiopia; an application of revised Bongaarts model. Reprod. Health.

[58] McCleary-Sills AW, Stoebenau K, Hollingworth G (2014). Understanding the Adolescent Family Planning Evidence Base.

[59] Bennett IM, Frasso R, Bellamy SL, Wortham S, Gross KS (2013). Pre-teen literacy and subsequent teenage childbearing in a US population. Contraception.

